# Risk of Appendicitis among Children with Different Piped Water Supply: A Nationwide Population-Based Study

**DOI:** 10.3390/ijerph15081601

**Published:** 2018-07-28

**Authors:** Hao-Ming Li, Shi-Zuo Liu, Ying-Kai Huang, Yuan-Chih Su, Chia-Hung Kao

**Affiliations:** 1 Department of Radiology, E-Da Hospital, I-Shou university, Kaohsiung 824, Taiwan; s851101@ym.edu.tw (H.-M.L.); ed106088@edah.org.tw (S.-Z.L.); 2 Department of Radiology, Kaohsiung Municipal Min-Sheng Hospital, Kaohsiung 802, Taiwan; yingkaih@gmail.com; 3 Management Office for Health Data, China Medical University Hospital, Taichung 404, Taiwan; njm0654@livemail.tw; 4 College of Medicine, China Medical University, Taichung 404, Taiwan; 5 Graduate Institute of Biomedical Sciences, School of Medicine, College of Medicine, China Medical University, Taichung 404, Taiwan; 6 Department of Nuclear Medicine and PET Center, China Medical University Hospital, Taichung 404, Taiwan; 7 Department of Bioinformatics and Medical Engineering, Asia University, Taichung 413, Taiwan

**Keywords:** child health, hygiene, appendicitis, piped water, epidemiology

## Abstract

Appendicitis is a common surgical condition for children. However, environmental effects, such as piped water supply, on pediatric appendicitis risk remain unclear. This longitudinal, nationwide, cohort study aimed to compare the risk of appendicitis among children with different levels of piped water supply. Using data from Taiwan Water Resource Agency and National Health Insurance Research Database, we identified 119,128 children born in 1996–2010 from areas of the lowest piped water supply (prevalence 51.21% to 63.06%) as the study cohort; additional 119,128 children of the same period in areas of the highest piped water supply (prevalence 98.97% to 99.63%) were selected as the controls. Both cohorts were propensity-score matched by baseline variables. We calculated the hazard ratios (HRs) and 95% confidence intervals (CIs) of appendicitis in the study cohort compared to the controls by Cox proportional hazards regression. The study cohort had a raised overall incidence rates of appendicitis compared to the control cohort (12.8 vs. 8.7 per 10,000 person-years). After covariate adjustment, the risk of appendicitis was significantly increased in the study cohort (adjusted HR = 1.46, 95% CI: 1.35, 1.58, *p* < 0.001). Subgroup and sensitivity analyses showed consistent results that children with low piped water supply had a higher risk of appendicitis than those with high piped water supply. This study demonstrated that children with low piped water supply were at an increased risk of appendicitis. Enhancement of piped water availability in areas lacking adequate, secure, and sanitized water supply may protect children against appendicitis.

## 1. Introduction

Appendicitis is the most common surgical condition for children, with an annual incidence of approximately 0.1% and cumulative incidence of 3.2% by age 20 [1,2]. However, for such a common disease, its etiology is not yet fully understood. Several hypotheses have been proposed, including luminal obstruction, infection, innate immunity, and adaptive immunity related to hygiene [3,4,5,6]. In the 1980s, Barker et al. reported that improvement in household amenities and piped water supply was followed by an increased rate of acute appendicitis, suggesting a ‘hygiene hypothesis’ that reduced early exposure to gut pathogens in children possibly altered the adaptive immunity with inappropriate or excessive inflammatory response leading to appendicitis [7,8]. However, there was no further evidence to validate their findings.

In contrast, piped water is considered an improved source and an indicator of water, sanitation, and hygiene (WASH), due to less contamination with pathogens during transport and household storage compared to non-piped water [9,10]. WASH has been proved as an effective measure to improve child health by reducing the risk of diarrhea, helminth infection, and environmental enteropathy [11,12,13], but evidence of WASH on pediatric appendicitis is limited. Only two studies since 1990 investigated the association between WASH and pediatric appendicitis [14,15], showing conflicting results compared to Barker’s studies. The effect of piped water supply on pediatric appendicitis risk remains uncertain. Therefore, we hypothesized that piped water availability in children was associated with the incidence of appendicitis, and conducted a longitudinal, nationwide cohort study to compare the risk of appendicitis among children with different levels of piped water supply. Our findings may help to develop strategies for preventing pediatric appendicitis.

## 2. Materials and Methods 

The design and reporting of this study followed STROBE (Strengthening the Reporting of Observational Studies in Epidemiology) guidelines [16] (Supplementary STROBE-checklist).

### 2.1. Data Source, Protocol Approvals, and Patient Consents

This retrospective cohort study uses Taiwan National Health Insurance (TNHI) database to assess the health outcomes of participants, and statistical data from Taiwan Water Resources Agency (TWRA) for the exposure of piped water supply. Both datasets were linked by the residential areas of participants. The TNHI program has covered 99.5% of the whole population in Taiwan since 1995 [17]. The TNHI database contains comprehensive medical claims data (clinic visit date, admission date, prescription, and operation records), disease status (diseases diagnosis), and demographics (sex, birthday, income, residential area) of each enrollee. Individual identifiers were scrambled and encrypted to protect personal privacy before released for research purpose. The dataset we used comprised the random sample of half children population (age < 18) among all residents in Taiwan. Taiwan‘s government constructed the universal piped water system for domestic water supply, with regular monitoring of supply quality and water usage by TWRA. This study was approved by the Research Ethics Committee of China Medical University and Hospital in Taiwan (CMUH-104- REC2-115 and CRREC-103-048) for exemption of informed consents.

### 2.2. Study Areas and Exposure Measurement

**Figure 1:**
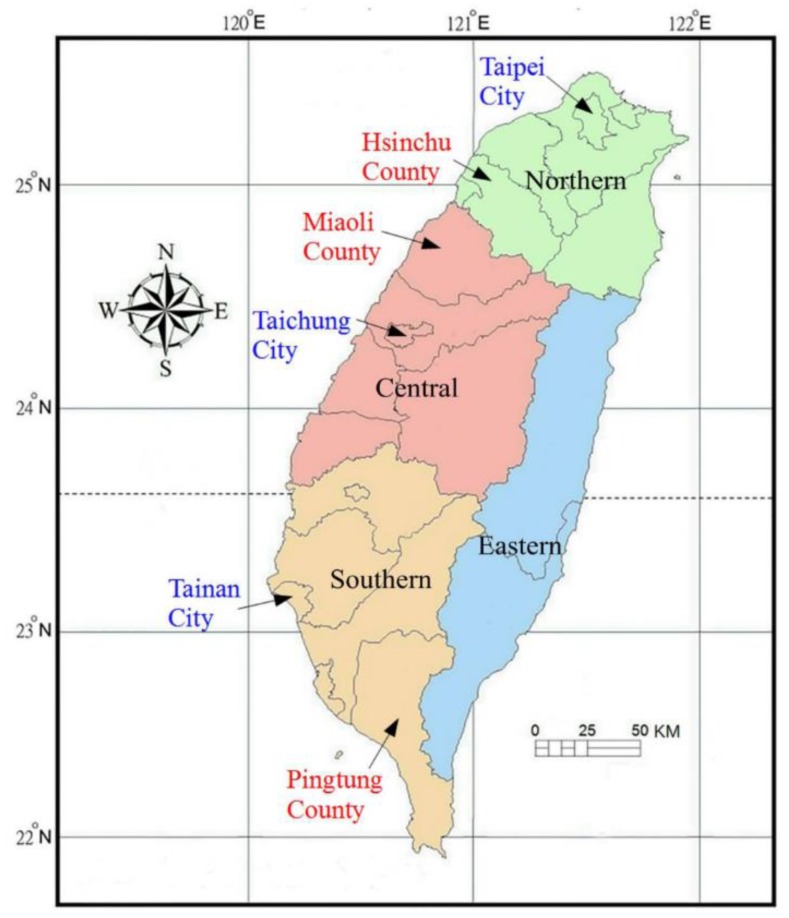
Study areas from the northern, central, and southern regions of Taiwan (red font: study group; blue font: control group).

We obtained yearly piped-water prevalences (PWPs) in Taiwan, defined as the population with piped water divided by the total population, at city/county level from 1996 to 2010 [18], ranging from 38.14–99.90% (Supplementary Table S1). The areas of piped water supply were classified into four geographic regions: the northern, central, southern, and eastern regions. Our study areas included six cities/counties (shown in Figure 1 and Table 1), which had the highest and the lowest PWPs in the northern region (Taipei City: PWP 99.40–99.69%; Hsinchu County: PWP 61.20–79.76%), the central region (Taichung City: PWP 97.03–99.30%; Miaoli County: PWP 65.15–76.04%), and the southern region (Tainan City: PWP 99.88–99.90%; Pingtung County: PWP 38.14–44.91%). The reasons for selecting these areas were as follows. (i) The PWPs were dynamically improved by years, and the PWP differences between most areas were small (less than 10%), which may lead to misclassification bias. Therefore, we chose the areas with the highest and lowest PWP for comparison (PWP differences over 20%), so that misclassification bias could be minimized. (ii) To avoid aggregation bias from clustering cases in a single sample area, we selected study areas from each region except the eastern region, because only two counties in the eastern region and their PWPs were similar.

### 2.3. Study Population and Period/ Covariates and Outcome Assessment

From the TNHI database, we identified children born in 1996–2010 with residential areas of Hsinchu County, Miaoli County, and Pingtung County as the study group, who had the lowest piped water supply. The control group consisted of children born in 1996–2010 with residential areas of Taipei city, Taichung City, and Tainan City, who had the highest piped water supply. The index date (the start of follow-up) of each participant was six months after their birthday when most infants were less breastfed and expected to increase water intake. All participants were followed from their index date until the first date of outcome, death, or the end of 2012 without missing data. Comorbidities before the index date, including gastrointestinal disorder, infectious diseases, perinatal disorder, low birth weight, or nutritional deficiency were identified by using the International Classification of Disease, Ninth Revision (ICD-9) codes with the algorithms validated in previous studies [19,20]. Geographic region and socioeconomic status of low-income were also added in covariate analysis. The low-income status was defined as low-income households unable to pay the TNHI premium that require government subsidies. The primary outcome was hospitalized appendicitis with a discharge ICD-9 code of 540-543 (appendicitis). Perforated appendicitis (ICD-9 540.0, 540.1) were also assessed. The case definition of appendicitis has been validated in numerous studies [1,2].

**Figure 2:**
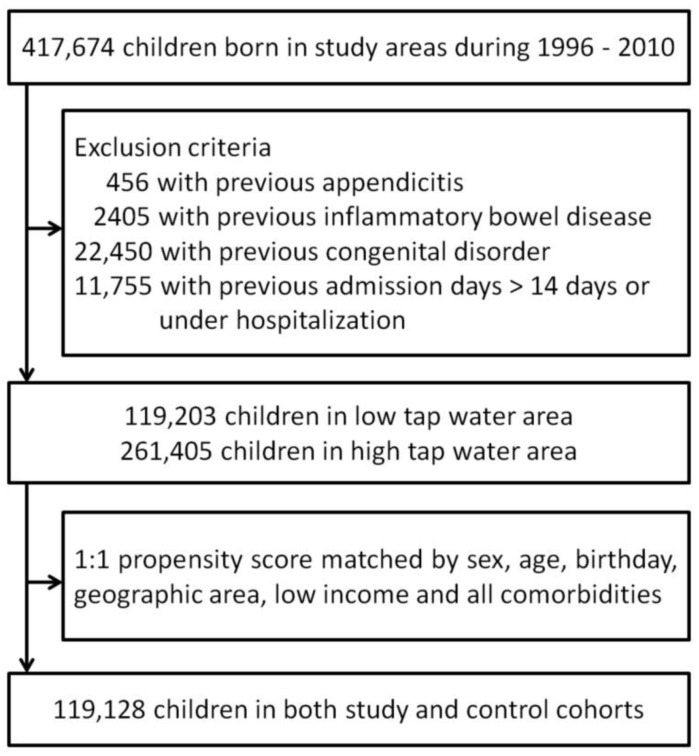
Patient selection diagram.

Exclusion criteria were: (i) appendicitis, congenital disorder and inflammatory bowel disease before index date; and (ii) hospitalization on index date or hospitalized days before index date >14 days, to avoid the surveillance bias of hospitalization and confoundings from disease severity. Supplementary Table S2 lists the ICD-9 codes for diseases and comorbidities defined in this paper. After the exclusion criteria, both cohorts were 1:1 propensity-score matched by sex, birth year, geographic region, low-income status, and all comorbidities. (Figure 2: patient selection diagram) Besides, the city areas in Taiwan had generally superior piped water coverage that the control group were all from cities, while the study group were all from counties. Therefore, we made an alternative control cohort from the highest PWP counties in each region (Taipei County: PWP 95.10–97.34%; Yunlin County: PWP 92.89–95.19%; Tainan County: PWP 96.83–98.12%), as sensitivity analysis to test if selection bias exists.

### 2.4. Statistical Analysis

We tested the differences in baseline characteristics between two cohorts by the standardized mean difference (SMD), while SMD > 0.1 indicates a meaningful imbalance of baseline variables [21]. We assessed the cumulative incidence of appendicitis in both groups by the Kaplan–Meier curves and tested their differences with the log-rank test. We calculated the incidence rate (per 10,000 person-years) of appendicitis in both cohorts through time-to-event data. We applied univariate Cox proportional hazards model to estimate crude hazard ratios (HRs) and 95% confidence intervals (CIs) of the appendicitis risk in the study cohort compared to the control, and multivariate analysis to calculate adjusted hazard ratios (aHRs) after controlling for all prior-selected covariates in Table 2. We also performed univariate and multivariate subgroup analyses of sex, birth year (in five-year strata), geographic regions, low-income status, and comorbidity. We verified the assumption of proportional hazards with graphical plotting method (*p* > 0.05). All analyses were done with the statistical software package, SAS, version 9.4 (SAS Institute, Inc., Cary, NC, USA), with a two-sided significance level of 0.05.

## 3. Results

**Figure 3:**
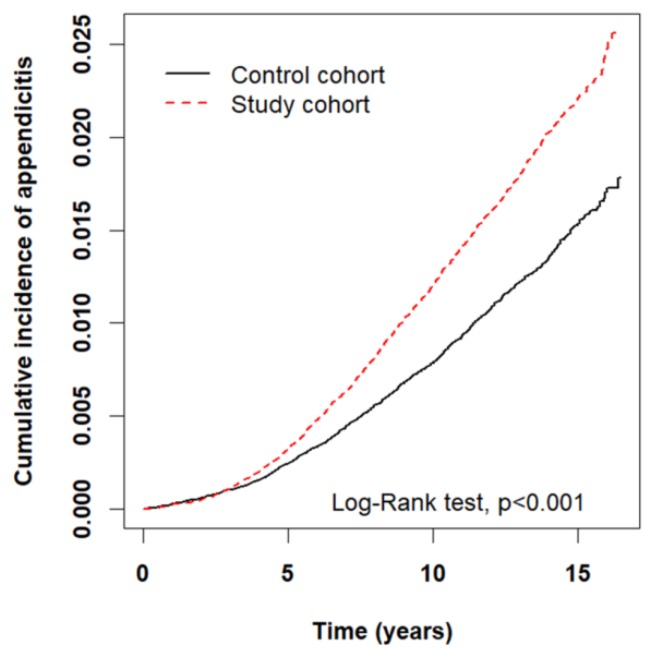
Cumulative incidence of appendicitis in both cohorts by Kaplan–Meier method.

Totally 238,256 patients were enrolled, with 119,128 patients in each cohort. There was no meaningful imbalance of baseline variables between both cohorts (SMD < 0.1, Table 2). The mean follow-up time was 10.1 years in the study cohorts and 10.0 years in the control cohort. The PWPs improved from 1996 to 2010 in both cohorts, where the improvement was more prominent in the study cohort about 12% (study cohort: 51.21% to 63.06%, control cohort: 98.97% to 99.63%, Table 1). The overall incidence rate of appendicitis was increased in the study cohort compared to the control cohort (12.8 vs. 8.7 per 10,000 person-years), reflecting an absolute excess risk of 4.1 cases per 10,000 person-years, with an aHR of 1.46 (95% CI: 1.35–1.58, *p* < 0.001, Table 3). In the subgroup analyses, most subgroups in the study cohort had higher relative risks of appendicitis than those in the control cohort. The difference of appendicitis risk between two cohorts was insignificant in the population born at 2006–2010 and with low income. The use of imaging modalities to diagnose appendicitis cases did not differ significantly between the study and control cohorts (data not shown, 29.6% vs. 32.9%, *p* > 0.05), so did the proportion of perforated appendicitis cases (23.06% vs. 21.35%, *p* > 0.05). But the study cohort had a consistently raised incidence rate of perforated appendicitis compared to the controls (2.95 vs. 1.86 per 10,000 person-years). Figure 3 shows that the study cohort had a significant cumulative incidence of appendicitis compared to the control cohort (*p* < 0.001).

### Association between Piped Water Supply and Pediatric Appendicitis

**Figure 4:**
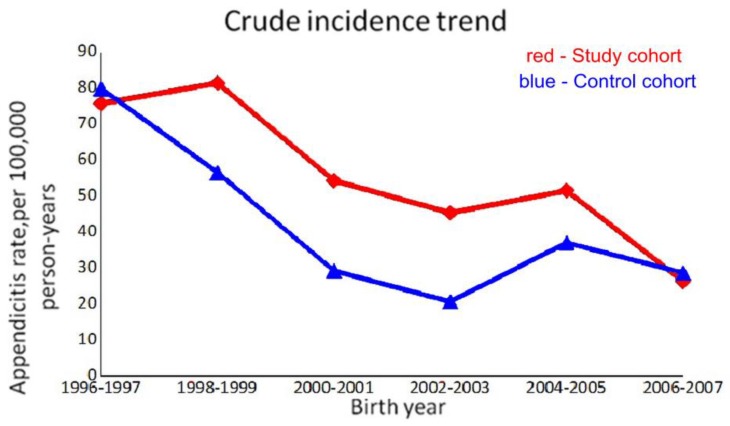
Trends of crude appendicitis incidence among 0–5 year children in both cohorts with birth year from 1996 to 2007.

In the multivariate analysis of pediatric appendicitis risk factors (Table 4), low piped water supply was a significant risk factor for pediatric appendicitis (aHR 1.46, 95% CI: 1.35–1.58, *p* < 0.001). Regional risk of pediatric appendicitis also existed in the southern and central regions compared to the northern region (southern: aHR 1.78, 95% CI:1.6–1.98; central: aHR 1.77, 95% CI: 1.59–1.98), with inversely related PWPs (southern: 65.17–70.70%; central: 84.60–91.36%; northern: 94.12–96.42%). We also found that children born in the later years had lower risk of appendicitis. Therefore, we analyzed the crude appendicitis incidence of 0–5 years old children with different birth-year in both cohorts (Figure 4), showing a trend of decreasing incidence from 1996 to 2007 as the PWPs improved in both cohorts. The sensitivity analyses with an alternative control cohort from counties showed similar results in children with low piped water supply (aHR 1.4, 95% CI: 1.29–1.51, *p* < 0.001, Supplementary Tables S3–S5).

## 4. Discussion

The present study shows that children living in areas of PWP less than 80% had a higher risk of appendicitis than those living in areas of PWP over 97%, with an absolute excess risk of 4.1 cases per 10,000 person-years (12.8 vs. 8.7 in Table 4). The estimated relative risks were significantly raised in almost all subgroup analyses, except for children born at 2006–2010 and with low-income status. The cases in these two subgroups were too few, that a small difference would affect the results. The relative risk increased in older birth cohorts, implying a possible dose effect or threshold effect of low piped water supply on pediatric appendicitis. We made a parallel comparison of children at the same period with different PWPs across areas (Table 4), and a longitudinal comparison of children in the same areas with different PWPs across years (Figure 4). Both showed consistent results that the incidence of pediatric appendicitis was inversely related to the PWP.

Our findings were compatible with previous studies that children lacking hygiene amenities, such as piped water supply and bathroom, had a raised risk of appendicitis [14,15]. In contrast, British studies at the 1980s found a raised rate of appendicitis following improved piped water supply (PWP from 49% to 80%), and a reduced risk of appendectomy in children living without hygiene amenities [7,8]. They proposed a hygiene hypothesis that improved hygiene reduced training on the adaptive immunity in young children, leading to their vulnerability to appendicitis when exposed to later infections in grown-ups. They also predicted that children with poor hygiene conditions in developing countries would have outbreaks of appendicitis when housing improves [7,8]. However, in the report from a recent systematic review of global appendicitis incidence, the annual incidence change was variable in developing countries, which did not support the prediction of hygiene hypothesis [22]. One possible explanation of these conflicting findings may be that the populations in the studies of Barker et al. were much different and older compared to ours (1932–1975 vs. 1996–2010 cohort; Britain vs. Taiwan) so that the nature of microbial exposure, subjects’ constitution, and regional risk factors differed. 

In Taiwan, the piped water system follows the drinking-water guidelines of WHO [23], with source protection, disinfection treatment, regular maintenance, and surveillance to prevent contamination. However, non-piped water sources such as underground and surface water are less protected and not treated, with susceptibility to fecal and waste contamination. A recent meta-analysis revealed that piped water was less likely to be contaminated compared with non-piped water at both the source and in household stored water [9]. In the water surveillance data from Environmental Protection Administration of Taiwan [24], the coliform counts in piped water were less than 1 CFU/100 mL, while the coliform counts in surface water were over 10^6^ CFU/100 mL. Besides, piped water had a much better quality of chemicals, metals, and dissolved solids because of the purification and treatment (Supplementary Table S6). Pathogenic *Escherichia coli (E. coli)* contamination of raw water in Taiwan was common, up to 31.8% [25]. Direct use of non-treated raw water may cause infection. Water-associated infections produce a heavy disease burden globally, including bacterial, viral, vector-based, and parasite infections [26,27]. Outbreaks of waterborne bacteria (like *Salmonella, Campylobacter, E. coli,*), viruses (like adenovirus), and parasites (like *Entamoeba, Schistosoma, Ascaris, Strongyloides* sp) have also been reported [12,13,26,27]. Furthermore, these pathogens could induce appendicitis [28]. To decrease the risk of these infections, a sanitized water supply had been proved as an effective intervention, especially in children [11,12,13]. The above could explain our findings and imply that prevention of infection may outweigh hygiene hypothesis to protect children against appendicitis nowadays [29].

This study has strengths of a large-scale nationwide population with minimal sampling error, a well-matched control group to adjust for confounders, longitudinal data with a high follow-up rate, and less information bias by case ascertainment of appendicitis from medical records rather than from questionnaire or interview. Some limitations exist in this study. First, information about diet, body mass index, smoking habits, household crowding, and genetics is lacking in TNHI database. These variables could not be adjusted in the analysis. However, smoking is rare and prohibited in children; effects of diet, household crowding, and genetics on appendicitis risk are under investigation without established conclusions. Second, piped water supply was part of urbanization process in Taiwan so that the areas with the highest PWPs were all cities and the areas with the lowest PWPs were all counties. The medical practice and utility may differ across cities and counties leading to confounding bias. To examine if appendicitis was diagnosed differently across regions, we scrutinized the medical records of appendicitis cases in both groups, showing no significant differences in the proportion of imaging diagnosis or perforated appendicitis related to delayed diagnosis. Besides, we performed sensitivity analyses with an alternative control cohort from counties of high PWPs, to test if unmeasured covariates between cities and counties led to selection bias. The results were consistent with previous analyses (Supplementary Tables S3–S5). Therefore, we believe selection bias was minimal in this study. Third, as an indicator of WASH, piped water supply is highly correlated with other WASH interventions, such as hand washing and toilet facility. We could not obtain the exposure information of other WASH interventions for further analysis. Nevertheless, it is difficult and unreasonable to separate the influence of other WASH interventions from piped water supply on pediatric appendicitis in this study. Fourth, migration of subjects might cause misclassification bias. However, from the data of Taiwan population census in 2010, the rate to move across county or city in aged 0–14 years was lowest about 3.6% [30]. Besides, the areas of study and control cohorts were not closely adjacent (Figure 1). Thus, migration of subjects is unlikely to affect our conclusion. Finally, this study is inherent to the limitation of ecological fallacy for the exposure data of piped water supply collected at group levels instead of individual levels. We could only find the inverse association between the risk of pediatric appendicitis and PWP, but unable to quantify a definite level of PWP that may prevent pediatric appendicitis in this study. The results of this study may be more applicable to public health policy than to personal risk prediction. Further studies with more accurate individual exposure of piped water supply should be conducted to clarify and quantify the effect of piped water supply on pediatric appendicitis.

## 5. Conclusions

This cohort study investigated the association between piped water supply and pediatric appendicitis in a nationwide population, showing that children with low piped water supply were at an increased risk of appendicitis. We proved such association through a parallel comparison across different areas at the same period, and a longitudinal comparison across years in the same areas. Our findings could provide new evidence to support WASH for child health improvement. The government and public should put efforts on the enhancement of piped water availability in areas lacking adequate, secure, and sanitized water supply, which may protect children against appendicitis.

## Figures and Tables

**Table 1:** Piped water prevalence in study areas during 1996–2010 (red font: study group; blue font: control group; unit: %).

Area\Year	1996	1997	1998	1999	2000	2001	2002	2003	2004	2005	2006	2007	2008	2009	2010
Northern Region	93.10	93.90	94.80	95.20	95.40	95.19	95.33	95.39	95.59	95.91	96.16	96.34	96.42	96.38	96.47
Taipei City	99.40	99.45	99.46	99.48	99.48	99.48	99.56	99.57	99.58	99.58	99.60	99.60	99.62	99.62	99.69
Hsinchu County	61.20	67.12	69.16	70.18	71.77	71.70	71.86	72.49	73.39	76.62	76.68	77.82	78.76	79.31	79.76
Central Region	85.70	86.40	86.50	86.70	86.80	87.19	87.63	87.81	88.40	88.73	89.09	89.39	89.47	89.48	89.60
Miaoli County	65.15	67.03	67.34	68.94	69.80	70.29	70.91	72.29	73.24	73.91	74.60	75.22	75.33	75.43	76.04
Taichung City	97.03	97.20	97.24	97.30	97.27	97.34	98.57	99.03	99.05	99.06	99.14	99.18	99.22	99.26	99.30
Southern Region	86.70	87.00	87.10	87.30	87.40	87.55	87.84	87.93	88.33	88.57	88.66	88.64	88.93	88.96	89.08
Tainan City	99.88	99.88	99.88	99.88	99.88	99.88	99.88	99.88	99.90	99.90	99.88	99.88	99.88	99.88	99.88
Pingtung County	38.14	38.49	40.89	41.55	41.69	41.67	42.07	42.67	43.66	44.02	44.28	43.67	45.13	44.98	44.91
Control group	98.97	99.24	99.06	99.07	99.06	99.06	99.39	99.50	99.51	99.51	99.54	99.55	99.57	99.58	99.63
Study group	51.21	51.71	55.06	56.14	56.88	57.04	57.52	58.42	59.47	60.71	61.17	61.46	62.50	62.70	63.06

**Table 2:** Baseline characteristics in the study and control cohorts.

Characteristics	Control, *n* (%)	Study, *n* (%)	SMD
**Sex**			0.006
Female	56,971 (47.8)	57,312 (48.1)	
Male	62,157 (52.2)	61,816 (51.9)	
**Birth year**			
1996–2000	51,883 (43.6)	52,843 (44.4)	0.016
2001–2005	39,034 (32.8)	39,544 (33.2)	0.009
2006–2010	28,211 (23.7)	26,741 (22.4)	0.029
**Geographic region**			0
North	35,966 (30.2)	35,966 (30.2)	
Central	35,195 (29.5)	35,195 (29.5)	
South	47,967 (40.3)	47,967 (40.3)	
**Low income**	648 (0.5)	808 (0.7)	0.017
**Comorbidity**			
Gastrointestinal disorder	3,7465 (31.4)	37,152 (31.2)	0.016
Infectious diseases	14,490 (12.2)	13,879 (11.7)	0.006
Perinatal disorder	9579 (8)	11,743 (9.9)	0.064
Low birth weight or nutritional deficiency	1389 (1.2)	960 (0.8)	0.036
Follow-up years, mean (SD)	10.0(4.21)	10.1(4.17)	

Abbreviation: SD, standard deviation; SMD, Standardized mean difference.

**Table 3:** Comparison of incidence and HR of appendicitis between study and control cohorts.

Variable	Control Cohort			Study Cohort			Compared with Control	
	Event ^a^	PY	Rate ^#^	Event ^a^	PY	Rate ^#^	Crude HR (95% CI)	Adjusted HR (95% CI) ^†^
**Total appendicitis**	1035	1,190,042	8.7	1535	1,199,483	12.8	1.47 (1.36–1.59) *	1.46 (1.35–1.58) *
**Perforated appendicitis**	221	1,190,042	1.86	354	1,199,483	2.95	1.59 (1.34–1.88) *	1.59 (1.34–1.88) *
**Sex**								
Female	418	570,791	7.32	633	577,563	11.0	1.49 (1.32–1.69) *	1.49 (1.32–1.69) *
Male	617	619,251	9.96	902	621,920	14.5	1.45 (1.31–1.61) *	1.44 (1.3–1.6) *
**Birth year**								
1996–2000	801	718,210	11.2	1167	728,223	16.0	1.44 (1.31–1.57) *	1.44 (1.31–1.57) *
2001–2005	196	352,496	5.56	332	357,496	9.29	1.67 (1.4–1.99) *	1.66 (1.39–1.98) *
2006–2010	38	119,335	3.18	36	113,764	3.16	0.99 (0.63–1.56)	1.03 (0.65–1.62)
**Geographic region**								
North	183	351,888	5.2	293	350,657	8.36	1.61 (1.34–1.93) *	1.61 (1.34–1.94) *
Central	348	353,217	9.85	507	350,390	14.5	1.47 (1.28–1.69) *	1.48 (1.29–1.69) *
South	504	484,937	10.4	735	498,436	14.8	1.41 (1.26–1.57) *	1.41 (1.26–1.58) *
**Low income**								
No	1033	1,186,587	8.71	1533	1,194,783	12.8	1.47 (1.36–1.59) *	1.46 (1.35–1.58) *
Yes	2	3454	5.79	2	4700	4.25	0.68 (0.09-4.83)	0.44 (0.06-3.49)
**Comorbidity**								
No	714	760,528	9.39	1018	756,664	13.45	1.43 (1.3–1.58) *	1.43 (1.3–1.58) *
Yes	321	429,514	7.47	517	442,819	11.68	1.55 (1.35–1.78) *	1.54 (1.34–1.76) *

Abbreviation: PY, person-years; HR, hazard ratio; CI, confidence interval; **^a^** Primary event: appendicitis (ICD-9 540-543); perforated appendicitis event assessed as (ICD-9 540.0, 540.1); ^†^ Adjusted for sex, birth year, geographic region, low income, and comorbidities; * *p* < 0.001; ^#^ Incidence rates, per 10,000 person-years.

**Table 4:** Multivariate analysis of risk factors for pediatric appendicitis.

Risk Factors	aHR ^†^	95% CI	*p*-Value
**Piped water prevalence (low vs. high)**	1.46	(1.35–1.58)	<0.001
**Sex (male vs. female)**	1.34	(1.24–1.45)	<0.001
**Birth year**			
1996–2000	1	(Reference)	
2001–2005	0.65	(0.59–0.73)	<0.001
2006–2010	0.47	(0.37–0.6)	<0.001
**Geographic region**			
North	1	(Reference)	
Central	1.77	(1.59–1.98)	<0.001
South	1.78	(1.6–1.98)	<0.001
**Low income (yes vs. no)**	0.73	(0.27–1.95)	0.53
**Comorbidity**			
Gastrointestinal disorder (yes vs. no)	1.04	(0.94–1.14)	0.50
Infectious diseases (yes vs. no)	1.09	(0.95–1.25)	0.22
Perinatal disorder (yes vs. no)	0.98	(0.85–1.12)	0.74
Low birth weight or nutritional deficiency (yes vs. no)	0.9	(0.58–1.39)	0.64

Abbreviation: aHR, adjusted hazard ratio; CI, confidence interval; ^†^ Adjusted for sex, birth year, geographic region, low income, and comorbidities.

## Supplementary Materials

The following are available online at http://www.mdpi.com/1660-4601/15/8/1601/s1, Table S1: Piped water prevalence in Taiwan during 1996–2010, Table S2: The ICD-9 codes for disease identification, Table S3: Baseline characteristics in the study and alternative control cohorts—sensitivity analysis, Table S4: Comparing the incidence and HR of appendicitis between the study and alternative control cohorts—sensitivity analysis, Table S5: Multivariate analysis of appendicitis risk factors—sensitivity analysis, Table S6: Average water quality in Taiwan during 2002–2011; STROBE checklist.

## Abbreviations

aHR	adjusted hazard ratio
CI	confidence interval
HR	hazard ratio
ICD-9	International Classification of Disease, Ninth Revision
PWP	piped-water prevalence
SMD	standardized mean difference
TNHI	Taiwan National Health Insurance
TWRA	Taiwan Water Resources Agency
WASH	water, sanitation, and hygiene
