# Supplementary material for: Risk of Appendicitis among Children with Different Piped Water Supply: A Nationwide Population-Based Study
Source: Int J Environ Res Public Health. 2018 Jul 28;15(8):1601. doi: 10.3390/ijerph15081601 (PMC6121532; doi:10.3390/ijerph15081601)
Supplement: Supplementary file 1 [file ijerph-15-01601-s001.pdf]

International Journal of
Environmental Research and Public Health

MDPI

Supplementary Materials

Evaluation the effect of piped water supply on pediatric appendicitis: a nationwide cohort study

Hao-Ming Li, Shi-Zuo Liu, Ying-Kai Huang, Yuan-Chih Su, and Chia-Hung Kao

Table S1. Piped water prevalence in Taiwan during 1996-2010

Area \ Year	1996	1997	1998	1999	2000	2001	2002	2003	2004	2005	2006	2007	2008	2009	2[illegible]
Northern Region	93.10	93.90	94.80	95.20	95.40	95.19	95.33	95.39	95.59	95.91	96.16	96.34	96.42	96.38	9[illegible]
**Taipei City**[#]	**99.40**	**99.45**	**99.46**	**99.48**	**99.48**	**99.48**	**99.56**	**99.57**	**99.58**	**99.58**	**99.60**	**99.60**	**99.62**	**99.62**	**9**[illegible]
Taipei County	95.10	95.11	96.07	96.33	96.52	96.68	96.69	96.87	96.93	97.09	97.16	97.21	97.25	97.29	9[illegible]
Keelung City	98.37	98.53	98.66	98.72	98.38	98.51	98.97	99.22	99.23	99.28	99.31	99.34	99.36	99.31	9[illegible]
Taoyuan County	88.80	90.87	92.33	93.66	93.89	92.46	93.01	92.69	93.20	93.69	94.65	95.15	95.11	94.67	9[illegible]
Hsinchu City	94.73	96.12	96.47	96.78	97.05	96.81	96.36	97.20	97.93	97.76	97.65	98.21	98.82	98.84	9[illegible]
**Hsinchu County***	**61.20**	**67.12**	**69.16**	**70.18**	**71.77**	**71.70**	**71.86**	**72.49**	**73.39**	**76.62**	**76.68**	**77.82**	**78.76**	**79.31**	**7**[illegible]
Yilan County	81.50	83.11	86.46	87.67	87.97	88.21	88.49	88.59	89.18	90.16	91.15	91.32	91.60	92.11	9[illegible]
Central Region	85.70	86.40	86.50	86.70	86.80	87.19	87.63	87.81	88.40	88.73	89.09	89.39	89.47	89.48	8[illegible]
**Miaoli County***	**65.15**	**67.03**	**67.34**	**68.94**	**69.80**	**70.29**	**70.91**	**72.29**	**73.24**	**73.91**	**74.60**	**75.22**	**75.33**	**75.43**	**7**[illegible]
**Taichung City**[#]	**97.03**	**97.20**	**97.24**	**97.30**	**97.27**	**97.34**	**98.57**	**99.03**	**99.05**	**99.06**	**99.14**	**99.18**	**99.22**	**99.26**	**9**[illegible]
Taichung County	81.70	82.28	82.98	82.36	82.34	83.16	83.65	84.13	85.14	85.83	86.54	87.01	87.20	87.24	8[illegible]
Nantou County	79.60	79.21	79.43	77.45	77.29	77.31	77.75	78.18	78.59	78.70	78.92	79.18	78.84	78.53	7[illegible]
Changhua County	88.40	89.22	89.31	90.50	90.59	91.07	90.96	90.71	91.18	91.58	91.95	92.30	92.32	92.31	9[illegible]
Yunlin County	92.89	95.10	95.19	95.19	95.19	94.98	94.96	93.72	94.15	93.79	93.54	93.40	93.57	93.60	9[illegible]
Southern Region	86.70	87.00	87.10	87.30	87.40	87.55	87.84	87.93	88.33	88.57	88.66	88.64	88.93	88.96	8[illegible]
Chiayi City	97.30	98.62	98.98	99.00	99.03	99.05	99.07	98.98	99.44	99.46	99.51	99.58	99.66	99.69	9[illegible]
Chiayi County	86.29	88.35	88.38	88.39	88.42	88.41	88.51	87.98	88.46	88.99	89.07	89.02	88.93	88.99	8[illegible]
**Tainan City**[#]	**99.88**	**99.88**	**99.88**	**99.88**	**99.88**	**99.88**	**99.88**	**99.88**	**99.90**	**99.90**	**99.88**	**99.88**	**99.88**	**99.88**	**9**[illegible]
Tainan County	97.68	96.83	97.06	97.13	97.12	97.42	97.76	97.75	97.78	97.79	97.80	97.92	97.95	98.01	9[illegible]
Kaohsiung City	97.28	95.95	98.15	98.09	98.23	98.61	98.86	98.89	98.93	98.96	99.00	99.04	99.06	99.07	9[illegible]

Kaohsiung County	88.54	88.62	88.63	88.62	88.51	88.48	88.69	88.87	89.62	90.20	90.08	90.03	90.19	90.26	[illegible]
**Pingtung County***	**38.14**	**38.49**	**40.89**	**41.55**	**41.69**	**41.67**	**42.07**	**42.67**	**43.66**	**44.02**	**44.28**	**43.67**	**45.13**	**44.98**	[illegible]
Eastern Region	76.00	77.00	77.70	78.30	78.60	78.65	78.43	78.91	79.48	80.16	80.55	80.75	81.09	81.12	[illegible]
Hualien County	76.40	77.93	78.91	79.90	80.45	80.46	80.32	81.13	81.63	82.22	82.55	82.69	82.93	82.97	[illegible]
Taitung County	75.22	75.73	75.86	75.96	75.97	76.04	75.69	75.70	76.37	77.17	77.62	77.90	78.38	78.40	[illegible]

* city/county in study group

# city/county in control group

Table S2. The ICD-9 codes for disease identification

**Disease**	**ICD-9 codes**
Appendicitis	540-543
- perforated appendicitis	540.0, 540.1
Congenital disorder	243, 250, 255.2, 270-273, 275, 277, 279, 343, 344, 740-759, V13.6
Gastrointestinal disorder	530-579
Infectious Diseases	001-139
Inflammatory bowel disease	555,556
Low birth weight	765, V213
Malignancy	140-208
Nutritional deficency	260-269
Perinatal disorder	760-779, V137

ICD-9-CM = International Classification of Diseases, Ninth Revision, Clinical Modification

Sensitivity analysis

Table S3. Baseline characteristics in the study and alternative control cohorts – Sensitivity analysis

Characteristics	Alternative control, n(%)	Study, n(%)	SMD
**Sex**			0.001
Female	56321 (48.2)	56325 (48.2)	
Male	60453 (51.8)	60449 (51.8)	
**Birth year**			
1996-2000	51213 (43.9)	51870 (44.4)	0.011
2001-2005	38652 (33.1)	38518 (33)	0.002
2006-2010	26909 (23)	26386 (22.6)	0.011
**Geographic area**			0.00
North	35965 (30.8)	35965 (30.8)	
Central	35196 (30.1)	35196 (30.1)	

South	45613 (39.1)	45613 (39.1)	
**Low income**	573 (0.5)	659 (0.6)	0.01
**Comorbidity**			
Gastrointestinal disorder	36551 (31.3)	36158 (31)	0.015
Infectious Diseases	14009 (12)	13441 (11.5)	0.007
Perinatal disorder	9734 (8.3)	9618 (8.2)	0.004
Low birth weight or nutritional deficiency	1113 (1)	809 (0.7)	0.029
Follow-up years, mean(SD)	10.1 (4.18)	10.1 (4.18)	

Abbreviation: SD, standard deviation; SMD, Standardized mean difference.

Table S4. Comparing the incidence and HR of appendicitis between the study and alternative control cohorts – Sensivity analysis

Variable	Alternative Control			Study cohort			Compared with Control	
	Event [a]	PY	Rate [#]	Event [a]	PY	Rate [#]	Crude HR (95% CI)	Adjusted HR (95% CI) [†]
**Total appendicitis**	998	1173983	8.5	1472	1176899	12.51	1.47(1.36-1.59)***	1.47(1.35-1.59)***
**Perforated appendicitis**	244	1173983	2.08	338	1176899	2.87	1.38(1.17-1.63)***	1.38(1.17-1.63)***
**Sex**								
Female	395	567001	6.97	608	568271	10.7	1.53(1.35-1.74)***	1.53(1.35-1.74)***
Male	603	606982	9.93	864	608628	14.2	1.43(1.29-1.58)***	1.42(1.28-1.58)***
**Birth year**								
1996-2000	708	709868	9.97	1120	716659	15.63	1.57(1.43-1.72)***	1.57(1.43-1.72)***
2001-2005	248	349636	7.09	317	347885	9.11	1.29(1.09-1.52)**	1.28(1.09-1.52)**
2006-2010	42	114479	3.67	35	112355	3.12	0.85(0.54-1.33)	0.85(0.54-1.33)
**Geographic region**								
North	250	351325	7.12	293	350657	8.36	1.17(0.99-1.39)	1.17(0.99-1.39)
Central	313	351337	8.91	507	350393	14.47	1.63(1.41-1.87)***	1.63(1.41-1.87)***
South	435	471322	9.23	672	475848	14.12	1.53(1.35-1.72)***	1.52(1.35-1.72)***
**Low income**								
No	997	1170982	8.51	1470	1172960	12.53	1.47(1.36-1.59)***	1.47(1.36-1.59)***
Yes	1	3001	3.33	2	3939	5.08	1.17(0.11-12.94)	0.88(0.08-10.04)
**Comorbidity**								
No	627	748896	8.37	1005	756412	13.29	1.59(1.44-1.75)***	1.58(1.43-1.75)***
Yes	371	425087	8.73	467	420487	11.11	1.27(1.11-1.46)***	1.27(1.11-1.46)***

Abbreviation: PY, person-years; HR, hazard ratio; CI, confidence interval

[a] Primary event: appendicitis (ICD-9 540-543); perforated appendicitis event assessed as (ICD-9 540.0, 540.1)

[†] Adjusted for sex, birth year, geographic region, low income and comorbidities

* $p < 0.05$; ** $p < 0.01$; *** $p < 0.001$; [#] Incidence rates, per 10,000 person-years

Table S5. Multivariate analysis of appendicitis risk factors – Sensivity analysis

Risk factors	aHR [†]	95% CI	p-value
**Tap water prevelance(low vs high)**	1.47	(1.35-1.59)	<0.001
**Sex(Male vs Female)**	1.36	(1.26-1.48)	<0.001
**Birth year**			
1996-2000	1.00	(Reference)	
2001-2005	0.75	(0.68-0.84)	<0.001
2006-2010	0.56	(0.44-0.72)	<0.001
**Geographic area**			
North	1.00	(Reference)	
Central	1.5	(1.34-1.67)	<0.001
South	1.45	(1.31-1.61)	<0.001
**Low Income (Yes vs No)**	0.63	(0.2-1.97)	0.43
**Comorbidity**			
Gastrointestinal disorder(Yes vs No)	0.98	(0.89-1.09)	0.74
Infectious Diseases(Yes vs No)	1.12	(0.98-1.29)	0.10
Perinatal disorder	1.19	(1.04-1.36)	0.01
Low birth weight or nutritional deficiency	0.95	(0.61-1.46)	0.80

Abbreviation: aHR, adjusted hazard ratio; CI, confidence interval

† Adjusted for sex, birth year, geographic region, low income, and comorbidities

Table S6. Average water quality in Taiwan during 2002-2011.

Test Items	Unit	piped	river	underground
Coliform	CFU/100mL	<1	1527760	NT
Free Chlorine Residual	mg/L	0.5051	NT	NT
Ammonia	mg/L	0.0004	3.5220	1.0491
Chloride	mg/L	3.7006	556.4699	617.9895
Nitrate	mg/L	0.6723	0.4073	2.1456
Sulfate	mg/L	9.7825	NT	158.8795
Arsenic	mg/L	<0.0001	0.0011	0.0067
Cadmium	mg/L	<0.0001	0.0004	0.0002
Chromium	mg/L	<0.0001	0.0017	0.0015
Copper	mg/L	0.0027	0.0145	0.0028
Iron	mg/L	0.0036	NT	1.6571
Lead	mg/L	0.0001	0.0048	0.0032
Manganese	mg/L	0.0010	0.0631	0.5421
Mercury	mg/L	<0.0001	0.0001	<0.0001
Nickel	mg/L	<0.0001	NT	0.0002
Selenium	mg/L	<0.0001	0.0011	0.0011
Zinc	mg/L	0.0010	0.0492	0.0206
Total Hardness	mg/L	90.094	NT	470.5593
Total Dissolved Solids	mg/L	43.129	162.1453	1601.4373

NT: not tested

STROBE checklist of items that should be included in reports of observational studies

	Item No.	Recommendation	Page No.	Relevant text from manuscript
**Title and abstract**	1	(*a*) Indicate the study's design with a commonly used term in the title or the abstract	1	This longitudinal, nationwide, cohort study
		(*b*) Provide in the abstract an informative and balanced summary of what was done and what was found	1	Children with low piped water supply were at an increased risk of appendicitis
**Introduction**				
Background/rationale	2	Explain the scientific background and rationale for the investigation being reported	2	The effect of piped water supply on pediatric appendicitis remains uncertain
Objectives	3	State specific objectives, including any prespecified hypotheses	2	we hypothesized that piped water availability in children was associated with the incidence of appendicitis, and conducted a longitudinal, nationwide cohort study to compare the risk of appendicitis among children with different levels of piped water supply.
**Methods**				
Study design	4	Present key elements of study design early in the paper	2	This retrospective cohort study uses Taiwan National Health Insurance (TNHI) database to assess the health outcomes of participants, and statistical data from Taiwan Water Resources Agency (TWRA) for the exposure of piped water supply.
Setting	5	Describe the setting, locations, and relevant dates, including periods of recruitment, exposure, follow-up, and data collection	2,3	From the TNHI database, we identified children born in 1996-2010 with residential areas of Hsinchu County, Miaoli County, and Pingtung County as the study group, who had the lowest piped water supply. The control group consisted of children born in 1996-2010 with residential areas of Taipei city, Taichung City, and Tainan City, who had the highest piped water supply. The index date (the start of follow-up) of each participant was six months after their birthday when most infants were less breastfed and expected to increase water intake. All participants were followed from their index date until the first date of outcome, death or the end of 2012 without missing data.
Participants	6	(*a*) *Cohort study*—Give the eligibility criteria, and the sources and methods of selection of participants.	3-4	From the TNHI database, we identified children born in 1996-2010 with residential areas of Hsinchu County, Miaoli County, and Pingtung County as the study group, who had the lowest piped water supply. The control group consisted of children born in 1996-2010 with residential areas of Taipei city, Taichung City, and Tainan City, who had the highest piped water supply. Exclusion criteria were: (i) appendicitis, congenital disorder and inflammatory bowel disease before index date; (ii) hospitalization on index date or hospitalized days before index date > 14 days.

		(*b*) *Cohort study*—For matched studies, give matching criteria and number of exposed and unexposed	4	both cohorts were 1:1 propensity-score matched by sex, age, birthday, geographic region, low-income status and all comorbidities.
Variables	7	Clearly define all outcomes, exposures, predictors, potential confounders, and effect modifiers. Give diagnostic criteria, if applicable	4	The primary outcome was hospitalized appendicitis with a discharge ICD-9 code of 540-543 (appendicitis). Perforated appendicitis (ICD-9 540.0, 540.1) were also assessed.
Data sources/ measurement	8*	For each variable of interest, give sources of data and details of methods of assessment (measurement). Describe comparability of assessment methods if there is more than one group	3-4	Comorbidities before the index date, including gastrointestinal disorder, infectious diseases, perinatal disorder, low birth weight or nutritional deficiency were identified by using the International Classification of Disease, Ninth Revision (ICD-9) codes with the algorithms validated in previous studies (Li et al., 2017; Sung et al., 2016). Geographic region and socioeconomic status of low-income were also added in covariate analysis. The low-income status was defined as households requiring a bailout from the government, which was registered in NHI database.
Bias	9	Describe any efforts to address potential sources of bias	2-4	(i) the PWPs were dynamically improved by years, and the PWP differences between most areas were small (less than 10%), which may lead to misclassification bias. Therefore, we chose the areas with the highest and lowest PWP for comparison (PWP differences over 20%), so that misclassification bias could be minimized; (ii) to avoid aggregation bias from clustering case in a single sample areas, we selected study areas from each region except the eastern region, because only two counties in the eastern region and their PWPs were similar. (page 2)<br>hospitalization on index date or hospitalized days before index date > 14 days, to avoid the surveillance bias of hospitalization and confoundings from disease severity.(page 4)<br>we made an alternative control cohort from the highest PWP counties in each region (Taipei County: PWP 95.10-97.34%; Yunlin County: PWP 92.89-95.19%; Tainan County: PWP 96.83-98.12%), as sensitivity analysis to test if selection bias exists.(page 4)
Study size	10	Explain how the study size was arrived at	5	Totally 238256 patients were enrolled, with 119128 patients in each cohort.
Quantitative variables	11	Explain how quantitative variables were handled in the analyses. If applicable, describe which groupings were chosen and why	4	We also performed univariate and multivariate subgroup analyses of sex, age (in 5-year strata), geographic regions, low-income status, and comorbidity.
Statistical methods	12	(a) Describe all statistical methods, including those used to control for confounding	4	We tested the differences in baseline characteristics between two cohorts by the standardized mean difference (SMD), while SMD > 0.1 indicates a meaningful imbalance of baseline variables. We assessed the cumulative incidence of appendicitis in both groups by the Kaplan–Meier curves and tested their differences with the log-rank test. We calculated the incidence rate (per 10000 person-years) of appendicitis for each cohort. We applied

				univariable Cox proportional hazards model to estimate crude hazard ratios (HRs) and 95% confidence intervals (CIs) of the appendicitis risk in the study cohort compared to the control, and multivariate analysis to calculate adjusted hazard ratios (aHRs) after controlling for all priori-selected covariates in Table 3.
		(b) Describe any methods used to examine subgroups and interactions	4	We also performed univariate and multivariate subgroup analyses of sex, age (in 5-year strata), geographic regions, low-income status, and comorbidity.
		(c) Explain how missing data were addressed	9	All participants were followed from their index date until the first date of outcome, death or the end of 2012 without missing data.
		(d) Cohort study—explain how loss to follow-up was addressed		No loss to follow-up
		(e) Describe any sensitivity analyses	4	we made an alternative control cohort from the highest PWP counties in each region (Taipei County: PWP 95.10-97.34%; Yunlin County: PWP 92.89-95.19%; Tainan County: PWP 96.83-98.12%), as sensitivity analysis to test if selection bias exists.
**Results**				
Participants	13*	(a) Report numbers of individuals at each stage of study—eg numbers potentially eligible, examined for eligibility, included in the study, completing follow-up, and analysed	4	Figure 2. patient selection diagram
		(b) Give reasons for non-participation at each stage	4	Figure 2. patient selection diagram
		(c) Consider use of a flow diagram	4	Figure 2. patient selection diagram
Descriptive data	14*	(a) Give characteristics of study participants (eg demographic, clinical, social) and information on exposures and confounders	5	Table 2**.** Baseline characteristics in the study and control cohorts s
		(b) Indicate number of participants with missing data		No participants with missing data
		(c) *Cohort study*—Summarise follow-up time	5	The mean follow-up time was 10.1 years in the study cohorts and 10.0 years in the control cohort.
Outcome data	15*	*Cohort study*—Report numbers of outcome events or summary measures over time	5	Table 3. Comparison of incidence and HR of appendicitis between study and control cohorts
Main results	16	(*a*) Give unadjusted estimates and, if applicable, confounder-adjusted estimates and their precision (eg, 95% confidence interval). Make clear which confounders were adjusted for and why they were included	5	Table 3. Comparison of incidence and HR of appendicitis between study and control cohorts
		(*b*) Report category boundaries when continuous variables were categorized	5	Table 2&3

		(*c*) If relevant, consider translating estimates of relative risk into absolute risk for a meaningful time period	5	The overall incidence rate of appendicitis was increased in the study cohort compared to the control cohort (12.8 vs.8.7 per 1,0000 person-years), reflecting an absolute excess risk of 4.1 cases per 10000 person-years,
Other analyses	17	Report other analyses done—eg analyses of subgroups and interactions, and sensitivity analyses	5-7	In the subgroup analyses, most subgroups in the study cohort had higher relative risks of appendicitis than those in the control cohort. The difference of appendicitis risk between two cohorts was insignificant in the population born at 2006-2010 and with low income. The use of imaging modalities to diagnose appendicitis cases did not differ significantly between the study and control cohorts (data not shown, 29.6% vs. 32.9%, p > 0.05), so did the proportion of perforated appendicitis cases (23.06% vs. 21.35%, p > 0.05). But the study cohort had a consistently raised incidence rate of perforated appendicitis compared to the controls (2.95 vs. 1.86 per 10000 person-years). Figure 3 shows that the study cohort had a significant cumulative incidence of appendicitis compared to the control cohort (p < 0.001).<br>In the multivariate analysis of pediatric appendicitis risk factors (Table 4), low piped water supply was a significant risk factor for pediatric appendicitis (aHR 1.46, 95% CI 1.35-1.58, p < 0.001). Regional risk of pediatric appendicitis also existed in the southern and central regions compared to the northern region (southern: aHR 1.78, 95% CI 1.6-1.98; central: aHR 1.77, 95% CI 1.59-1.98), with inversely related PWPs (southern: 65.17-70.70%; central: 84.60-91.36%; northern: 94.12-96.42%). Besides, we found that children born in the later years had lower risk of appendicitis. Therefore, we analyzed the crude appendicitis incidence of 0-5 years old children with different birth-year in both cohorts (Figure 4), showing a trend of decreasing incidence from 1996 to 2007 as the PWPs improved in both cohorts. The sensitivity analyses with an alternative control cohort from counties showed similar results in children with low piped water supply (aHR 1.4, 95% CI 1.29-1.51, p < 0.001, Supplementary Table S3-S5).
**Discussion**				
Key results	18	Summarise key results with reference to study objectives	7	The present study shows that children living in areas of PWP less than 80% had a higher risk of appendicitis than those living in areas of PWP over 97%, with an absolute excess risk of 4.1 cases per 10000 person-years.
Limitations	19	Discuss limitations of the study, taking into account sources of potential bias or imprecision. Discuss both direction and magnitude of any potential bias	8-9	Some limitations exist in this study. First, information about diet, body mass index, smoking habits, household crowding, and genetics is lacking in TNHI database. These variables could not be adjusted in the analysis. However, smoking is rare and prohibited in children; effects of diet, household crowding, and genetics on appendicitis risk are under investigation without established conclusions. Second, piped water supply was part of urbanization process in

				Taiwan so that the areas with the highest PWPs were all cities and the areas with the lowest PWPs were all counties. The medical practice and utility may differ across cities and counties leading to confounding bias. To examine if appendicitis was diagnosed differently across regions, we scrutinized the medical records of appendicitis cases in both groups, showing no significant differences in the proportion of imaging diagnosis or perforated appendicitis related to delayed diagnosis. Besides, we performed sensitivity analyses with an alternative control cohort from counties of high PWPs, to test if unmeasured covariates between cities and counties led to selection bias. The results were consistent with previous analyses (Supplementary Table S3-S5). Therefore, we believe selection bias was minimal in this study. Third, as an indicator of WASH, piped water supply is highly correlated with other WASH interventions, such as hand washing and toilet facility. We could not obtain the exposure information of other WASH interventions for further anaylsis. Nevertheless, it is difficult and unreasonable to separate the influence of other WASH interventions from piped water supply on pediatric appendicitis in this study. Fourth, migration of subjects might cause misclassification bias. However, from the data of Taiwan population census in 2010, the rate to move across county or city in aged 0-14 years was lowest about 3.6% [30]. Besides, the areas of study and control cohorts were not closely adjacent (figure 1). Thus, migration of subjects is unlikely to affect our conclusion. Finally, this study is inherent to the limitation of ecological fallacy for the exposure data of piped water supply collected at group levels instead of individual levels. We could only find the inverse association between the risk of pediatric appendicitis and PWP, but unable to quantify a definite level of PWP that may prevent pediatric appendicitis in this study. The results of this study may be more applicable to public health policy than to personal risk prediction.
Interpretation	20	Give a cautious overall interpretation of results considering objectives, limitations, multiplicity of analyses, results from similar studies, and other relevant evidence	7	the incidence of pediatric appendicitis was inversely related to the PWP. Our findings were compatible with previous studies that children lacking hygiene amenities, such as piped water supply and bathroom, had a raised risk of appendicitis (Coggon et al., 1991; Gardikis et al., 2011).
Generalisability	21	Discuss the generalisability (external validity) of the study results	7	the incidence of pediatric appendicitis was inversely related to the PWP. Our findings were compatible with previous studies that children lacking hygiene amenities, such as piped water supply and bathroom, had a raised risk of appendicitis (Coggon et al., 1991; Gardikis et al., 2011).
**Other information**				
Funding	22	Give the source of funding and the role of the funders for the present study and, if applicable, for the original study	9	Taiwan Ministry of Health and Welfare Clinical Trial Center (MOHW106-TDU-B-212-113004), China Medical University Hospital, Academia

on which the present article is based	Sinica Taiwan Biobank Stroke Biosignature Project (BM10501010037), NRPB Stroke Clinical Trial Consortium (MOST 105-2325-B-039-003), Tseng-Lien Lin Foundation, Taichung, Taiwan, Taiwan Brain Disease Foundation, Taipei, Taiwan, and Katsuzo and Kiyo Aoshima Memorial Funds, Japan. The funders had no role in the study design, data collection and analysis, decision to publish, or preparation of the manuscript. No additional external funding was received for this study.

*Give information separately for cases and controls in case-control studies and, if applicable, for exposed and unexposed groups in cohort and cross-sectional studies.

Note: An Explanation and Elaboration article discusses each checklist item and gives methodological background and published examples of transparent reporting. The STROBE checklist is best used in conjunction with this article (freely available on the Web sites of PLoS Medicine at http://www.plosmedicine.org/, Annals of Internal Medicine at http://www.annals.org/, and Epidemiology at http://www.epidem.com/). Information on the STROBE Initiative is available at www.strobe-statement.org.
